# Distribution of Gastrointestinal and Dietary Risk Factors Among U.S. Adults Classified as Having Iron Deficiency Anemia Across Diagnostic Thresholds

**DOI:** 10.1002/ajh.70388

**Published:** 2026-05-30

**Authors:** Omar Al Ta’ani, Bridget M. Mayrer, Nicole M. Luche, Shazia Mehmood Siddique, Scott A. Peslak, Timothy S. Anderson, Ravy K. Vajravelu

**Affiliations:** ^1^ Department of Internal Medicine Allegheny Health Network Pittsburgh Pennsylvania USA; ^2^ Division of Gastroenterology, Hepatology and Nutrition, Department of Medicine University of Pittsburgh School of Medicine Pittsburgh Pennsylvania USA; ^3^ Division of General Internal Medicine, Department of Medicine University of Pittsburgh School of Medicine Pittsburgh Pennsylvania USA; ^4^ Division of Gastroenterology and Hepatology, Department of Medicine University of Pennsylvania Perelman School of Medicine Philadelphia Pennsylvania USA; ^5^ Division of Hematology/Oncology, Department of Medicine University of Pennsylvania Perelman School of Medicine Philadelphia Pennsylvania USA; ^6^ Center for Healthcare Evaluation, Research, and Promotion, VA Pittsburgh Healthcare System Pittsburgh Pennsylvania USA

**Abbreviations**
AGA: American Gastroenterological Association
ASH: American Society of Hematology
CI: confidence interval
IDA: iron deficiency anemia
IQR: interquartile range
NHANES: National Health and Nutrition Examination Survey
NSAID: nonsteroidal anti‐inflammatory drug
WHO: World Health Organization

To the Editor,

1

In 2020, the American Gastroenterological Association (AGA) published clinical management guidelines that sought to standardize iron deficiency anemia (IDA) management [1]. These guidelines recommend that men and postmenopausal women with asymptomatic IDA undergo esophagogastroduodenoscopy and colonoscopy (bidirectional endoscopy) and conditionally recommend the same management for premenopausal women with asymptomatic IDA. Additionally, in order to enhance sensitivity, these guidelines liberalized the diagnostic threshold for iron deficiency from serum ferritin ≤ 15 ng/mL, as recommended by the World Health Organization (WHO), to ≤ 45 ng/mL [1, 2, 3]. Furthermore, in draft guidelines released in September 2025, the American Society of Hematology (ASH) suggested a liberalized ferritin threshold at an intermediate value of ≤ 30 ng/mL based on low certainty of evidence [4].

We recently reported that compared with the ferritin ≤ 15 ng/mL threshold, the ≤ 30 and ≤ 45 ng/mL thresholds increase the number of individuals in the United States classified as having IDA by 2.4 and 3.3 million, respectively [5]. However, the impact of these new ferritin thresholds on the distribution of iron deficiency risk factors among individuals with IDA is unknown. To assess this impact, we conducted a cross‐sectional study utilizing data from the 2017–2020 cycle of the National Health and Nutrition Examination Survey (NHANES), which is the first nationwide survey in the United States to assess iron status among premenopausal women, postmenopausal women, and men since the 1990s. Using these data, we characterized gastrointestinal and dietary risk factors for iron deficiency among individuals classified as having IDA across ferritin thresholds from the WHO, ASH, and the AGA (full methodology in Supporting Information S1). NHANES protocols and analyses are approved by the National Center for Health Statistics Ethics Review Board [6]. All raw data are freely available via the National Center for Health Statistics at https://www.cdc.gov/nchs/nhanes.

We identified 7357 nonpregnant adults, representing 213.7 million (95% confidence interval, CI 195.5–232.0 million) individuals in the U.S. population. Of these, 58.2 million were premenopausal women (95% CI 51.2–65.3 million), 55.8 million were postmenopausal women (95% CI 50.0–61.6 million), and 99.7 million were men (95% CI 91.1–108.4 million). Demographic and health characteristics of these individuals are presented in Tables 1 and S1. Overall, 5.9 (95% CI 4.7–7.1), 8.3 (95% CI, 6.8–9.9), and 9.2 million (95% CI 7.6–10.7 million) individuals were classified as having IDA at the ferritin ≤ 15, ≤ 30, and ≤ 45 ng/mL thresholds, respectively (Table 2). At the ferritin ≤ 30 ng/mL threshold, 1.3 million (57%) individuals newly classified to have IDA were premenopausal women. At the ferritin ≤ 45 ng/mL threshold, 1.7 million (53%) individuals newly classified to have IDA were premenopausal women.

**TABLE 1:** Characteristics of nonpregnant adults from NHANES.

	All nonpregnant adults	Premenopausal women	Postmenopausal women	Men
Unweighted, *n*	7357	1901	2039	3417
Weighted, *n* (95% CI)	213.7 million (195.5–232.0 million)	58.2 million (51.2–65.3 million)	55.8 million (50.0–61.5 million)	99.7 million (91.0–108.4 million)
Demographics, % (95% CI) unless otherwise specified				
Female	53.3 (51.6–55.1)	100.0 (100.0–100.0)	100.0 (100.0–100.0)	
Male	46.7 (44.9–48.4)			100.0 (100.0–100.0)
Age, median (IQR)	48 (33–62)	35 (27–43)	63 (56–72)	46 (32–61)
Race				
Mexican American	8.4 (6.3–11.2)	11.6 (8.3–15.9)	4.4 (3.3–5.9)	8.8 (6.6–11.7)
Non‐Hispanic Asian	5.4 (3.9–7.4)	6.7 (4.7–9.4)	5.0 (3.5–7.2)	4.9 (3.5–6.7)
Non‐Hispanic Black	10.7 (8.2–13.9)	12.7 (10.1–16.0)	10.1 (7.2–14.1)	9.9 (7.6–12.8)
Non‐Hispanic White	63.8 (58.7–68.6)	56.7 (50.1–63.0)	70.5 (65.3–75.2)	64.2 (59.2–69.0)
Other Hispanic	7.6 (6.2–9.3)	8.5 (6.7–10.6)	6.6 (5.1–8.4)	7.8 (6.2–9.7)
Other race/multiracial	4.0 (3.3–4.8)	3.8 (2.8–5.2)	3.4 (2.2–5.3)	4.5 (3.8–5.3)
Covered by health insurance	86.6 (83.8–89.0)	85.3 (81.8–88.3)	93.4 (91.7–94.8)	83.6 (80.2–86.4)
Routine place to go for healthcare	83.5 (81.1–85.6)	82.8 (79.0–86.0)	94.2 (92.3–95.7)	77.8 (75.0–80.4)
Medical history and comorbidities, % (95% CI)				
History of blood transfusion	10.1 (9.0–11.2)	6.0 (4.5–8.0)	19.0 (16.4–22.0)	7.5 (6.3–8.8)
Advance fibrosis/cirrhosis, congestive heart failure, or chronic kidney disease	6.0 (5.1–6.9)	2.6 (1.9–3.6)	8.2 (6.4–10.3)	6.7 (5.9–7.6)
Anticoagulant, antiplatelet, aspirin, or NSAID use	18.6 (16.5–20.7)	4.1 (3.0–5.6)	28.9 (25.3–32.6)	21.2 (18.5–24.3)

**TABLE 2:** Absolute counts (millions, 95% CI) of IDA risk factors among individuals classified as having IDA stratified by serum ferritin threshold.

Risk factor	Ferritin ≤ 15 ng/mL (WHO)	Ferritin ≤ 30 ng/mL (ASH)	Ferritin ≤ 45 ng/mL (AGA)
All individuals with IDA	5.9 (4.7–7.1)	8.3 (6.8–9.9)	9.2 (7.6–10.7)
Gastrointestinal bleeding risk factors	0.7 (0.4–1.0)	1.4 (0.9–2.0)	1.7 (1.1–2.3)
Abdominal pain without gastrointestinal bleeding risk factors	1.1 (0.6–1.6)	1.8 (1.2–2.3)	1.9 (1.3–2.5)
Neither gastrointestinal bleeding risk factors nor abdominal pain	4.0 (3.1–5.0)	5.0 (3.8–6.1)	5.4 (4.2–6.5)
Not eligible for average‐risk colorectal cancer screening (ages 20–44)	3.3 (2.4–4.1)	4.3 (3.3–5.3)	4.7 (3.7–5.8)
Eligible for average‐risk colorectal cancer screening (ages 45 or older)	2.6 (1.7–3.5)	4.0 (2.9–5.1)	4.4 (3.3–5.6)
Sufficient dietary iron intake	1.7 (1.0–2.4)	3.1 (2.0–4.1)	3.5 (2.3–4.6)
Insufficient dietary iron intake	4.1 (2.7–5.5)	5.0 (3.5–6.4)	5.6 (4.2–7.0)

We assessed gastrointestinal bleeding risk factors as comorbidities and prescription medications associated with increased risk of gastrointestinal hemorrhage. At the ferritin ≤ 15 ng/mL threshold, 0.7 million individuals (95% CI 0.4–1.0 million) with IDA had gastrointestinal bleeding risk factors (Table 2). Liberalizing the ferritin threshold to ≤ 30 ng/mL did not statistically significantly increase the total number of individuals with gastrointestinal bleeding risk factors classified as having IDA (0.7 million, 95% CI 0.0–1.2 million), but liberalizing the ferritin threshold to ≤ 45 ng/mL did (1.0 million, 95% CI 0.3–1.8 million). In subgroup analyses, both thresholds classified more postmenopausal women and men with gastrointestinal bleeding risk factors as having IDA compared with the ≤ 15 ng/mL threshold (incremental counts in Figure 1 and total counts in Figure S1). At the ferritin ≤ 15 ng/mL threshold, 1.1 million (95% CI 0.6–1.6 million) individuals had abdominal pain without gastrointestinal bleeding risk factors, and the ≤ 30 and ≤ 45 ng/mL thresholds respectively classified an additional 0.7 (0.1–1.4) and 0.8 million (0.2–1.6 million) individuals with abdominal pain without gastrointestinal bleeding risk factors as having IDA.

**FIGURE 1:**
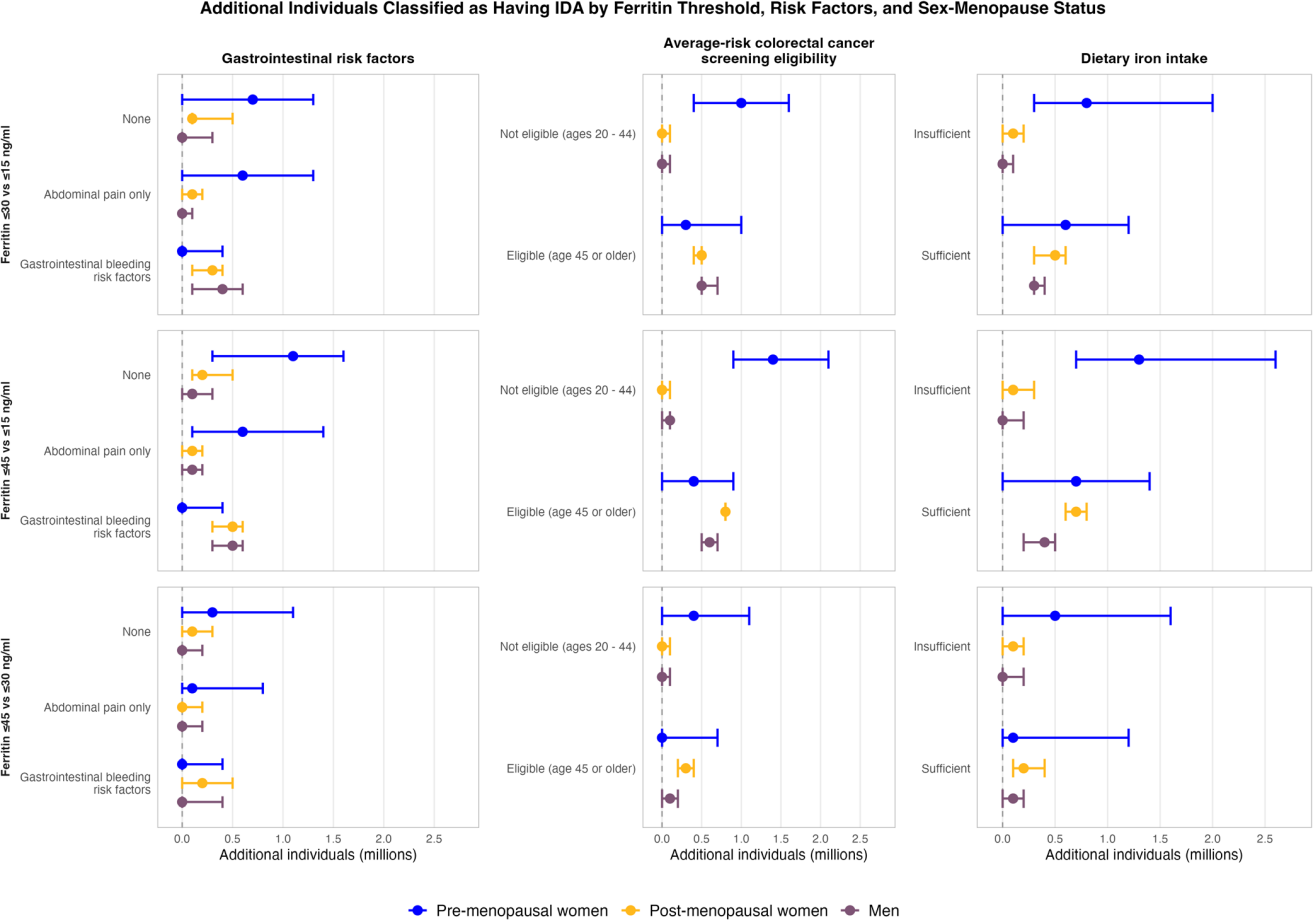
Additional individuals classified as having iron deficiency anemia by ferritin threshold, risk factors, and sex‐menopause status. Error bars denote 95% CIs, which were determined by bootstrapping. Gastrointestinal bleeding risk factors were congestive heart failure, chronic kidney disease, advanced hepatic fibrosis/cirrhosis, and anticoagulant, antiplatelet, aspirin, and nonsteroidal anti‐inflammatory drug use. Abdominal pain was ascertained from the medical questionnaire and indicates that the participant had nonmenstrual abdominal pain within the past 12 months. Individuals age 45 and older are eligible for average‐risk colorectal cancer screening [7, 8]. Dietary iron intake was calculated from the average of the day one and day two 24‐h dietary recall interviews plus any iron supplementation. Among nonpregnant adults, median daily iron intake was 11.7 mg (IQR 8.8–16.3) for premenopausal women (dietary reference Intake 18 mg), 11.3 mg (IQR 8.3–15.7) for postmenopausal women (Dietary Reference Intake 8 mg), and 14.6 mg (IQR 10.6–19.9) for men (dietary reference intake 8 mg).

Because occult gastrointestinal bleeding could be caused by undiagnosed colorectal cancer, we assessed the age distribution of individuals with IDA relative to guideline recommended screening age [7, 8]. At the ferritin ≤ 15 ng/mL threshold, 3.3 million individuals (95% CI 2.4–4.1 million) with IDA were aged 20–44 years, and 2.6 million (95% CI 1.7–3.5 million) were aged 45 years or older (Table 2). Liberalizing the threshold to ≤ 30 and ≤ 45 ng/mL increased the number of premenopausal women aged 20–44 years classified as having IDA by 1.0 (95% CI 0.4–1.6) and 1.4 million (95% CI 0.9–2.1 million), respectively (Figures 1 and S2).

With respect to dietary iron intake, at the ferritin ≤ 15 ng/mL threshold, 4.1 million individuals with IDA (95% CI 2.7–5.5 million), including 3.8 million (95% CI 2.3–5.3 million) premenopausal women, had insufficient daily dietary iron intake (Table 2). Liberalizing the threshold to ≤ 30 and ≤ 45 ng/mL increased the number of premenopausal women with insufficient daily dietary iron intake classified as having IDA by 0.8 million (95% CI 0.3–2.0) and 1.3 million (95% CI 0.7–2.6 million), respectively (Figures 1 and S3).

In summary, this nationally representative cross‐sectional study demonstrates that the AGA's recommendation to liberalize the serum ferritin threshold used to diagnose iron deficiency from ≤ 15 to ≤ 45 ng/mL classifies 0.8 million more postmenopausal women and 0.6 million more men as having IDA, and that most of these individuals have conditions associated with gastrointestinal iron loss. This indicates that a liberalized ferritin threshold has the potential to identify individuals with pathological iron deficiency risk factors earlier in their disease course. However, identifying these individuals is at the expense of classifying 1.7 million more premenopausal women as having IDA, with 76% of these individuals having insufficient dietary iron intake that could be treated with iron supplementation. Coupled with a recommendation from the AGA to pursue bidirectional endoscopy for patients with asymptomatic IDA, these findings indicate that liberalizing the threshold to diagnose iron deficiency from ferritin ≤ 15 to ≤ 45 ng/mL could lead to over‐utilization of endoscopy and patient burden, especially for premenopausal women. Additionally, we demonstrate that the ferritin ≤ 30 ng/mL threshold being considered by ASH results in similar distributions of iron deficiency risk factors among individuals classified as having IDA compared with the AGA's ≤ 45 ng/mL threshold.

Because there may be benefits to diagnosing early or subclinical IDA among premenopausal women, guidance that more precisely matches management strategies with IDA risk factors is needed. In the current guidelines, the AGA recommends that management decisions should be based on shared decision making. Unfortunately, there are few reliable estimates of the yield of bidirectional endoscopy for identifying the etiology of iron deficiency to guide these discussions as the existing evidence is mostly derived from small institutional case series or clinical settings that do not reflect U.S. gastrointestinal management [3, 9, 10, 11, 12, 13, 14]. An additional barrier to IDA shared decision making is that it is difficult and time‐consuming to accurately identify nongastrointestinal iron deficiency risk factors in clinical practice. For example, validated methods to diagnose insufficient dietary iron intake, such as food frequency questionaries and dietary recalls are rarely conducted in clinical practice because they take at least 1 h to administer and evaluate [15]. Additionally, diagnosing menorrhagia is difficult to operationalize because subjective report of heavy menstrual bleeding has low sensitivity and specificity and pictorial charts are not accurate when completed during an office visit [16, 17, 18, 19]. Novel clinical tools that quickly and accurately identify IDA risk factors are urgently needed. Together, these considerations identify high‐yield areas for future research that could enable risk‐stratified IDA diagnosis and management guidelines that allocate healthcare resources better than current recommendations.

This study has limitations. First, we could not assess trends in IDA risk factors because the 2017–2020 NHANES cycle is the only survey that contains ferritin data for postmenopausal women and men since 1999–2002. This also limited the precision of some of the subgroup estimates. Second, because NHANES does not contain data about hematochezia, we assumed that all individuals with iron deficiency did not have overt gastrointestinal bleeding that would disqualify them from the AGA guideline, which focuses only on asymptomatic patients. Third, some risk factors could have been misclassified. For example, we could have underestimated nonsteroidal anti‐inflammatory drug (NSAID) use because NHANES does not capture over‐the‐counter NSAIDs, and we could have overestimated dietary iron sufficiency because NHANES does not record whether participants consume vegetarian diets. Furthermore, we used broad conditions such as congestive heart failure as proxies for more specific conditions that are not identifiable in NHANES but are strongly associated with gastrointestinal bleeding, like aortic stenosis. Since these specific conditions are subsets of the broad proxy conditions we used in this study, we expect that the counts of patients with gastrointestinal risk factors reported in this study are upper bounds. Fourth, we used age as a proxy for undiagnosed colorectal cancer as a potential cause of IDA. More direct data about each participant's colorectal cancer screening history could provide better risk assessment. However, prior colorectal cancer screening history is not currently a modifying factor in any IDA management guideline. Future research should assess prior endoscopic evaluation as a potential modifying factor for IDA management.

In conclusion, this study provides epidemiological insights into how liberalizing the threshold for diagnosing iron deficiency to ≤ 30 or ≤ 45 ng/mL impacts the distribution of risk factors among individuals with IDA. Compared with a ≤ 15 ng/mL threshold, liberalized ferritin thresholds successfully identify more postmenopausal women and men with gastrointestinal risk factors for iron deficiency but also identify more premenopausal women without any gastrointestinal risk factors for IDA who have insufficient dietary iron intake. These findings lay a foundation for future research that determines IDA diagnosis, evaluation, and treatment best practices, which in turn could inform new risk‐stratified IDA guidelines.

## Author Contributions

Conceptualization: Al Ta'ani, Vajravelu. Methodology: Al Ta'ani, Mayrer, Vajravelu. Formal analysis: Al Ta'ani, Mayrer, Vajravelu. Investigation: all authors. Resources: Vajravelu. Data curation: Al Ta'ani, Mayrer, Vajravelu. Writing – original draft: Al Ta'ani, Vajravelu. Writing – review and editing: all authors. Visualization: Mayrer, Vajravelu. Supervision: Vajravelu. Project administration: Vajravelu. Funding acquisition: Vajravelu. The corresponding author attests that all listed authors meet authorship criteria and that no others meeting the criteria have been omitted.

## Funding

This work was supported by The National Institutes of Health/National Institute of Diabetes and Digestive and Kidney Diseases (Shazia M. Siddique, grant number K08‐DK120902 and Scott A. Peslak, grant number K08‐DK129716) and also by NIH/National Institute on Aging (Timothy S. Anderson, grant number K76‐AG074878).

## Conflicts of Interest

N.M.L., T.S.A., and R.K.V. are employees of the Department of Veterans Affairs. This research does not necessarily represent the views of the Department of Veterans Affairs or the United States Government. S.A.P. reports receiving research grants from the American Society of Hematology (ASH Scholar Award) and Blueprint Medicines—both unrelated to this research. T.S.A. reports receiving research grants from the Centers for Disease Control and Prevention, Department of Veterans Affairs, American Heart Association, and the US Deprescribing Research Network—all unrelated to this research. He also reports receiving personal fees from the American Medical Association unrelated to this research. R.K.V. reports consulting fees from the American Gastroenterological Association, paid to the University of Pittsburgh, outside the scope of the submitted work.

## Supporting information


**Table S1:** Unweighted counts.
**Figure S1:** Gastrointestinal risk factors among nonpregnant adults with iron deficiency anemia stratified by sex and menopausal status.
**Figure S2:** Age distribution among nonpregnant adults with iron deficiency anemia.
**Figure S3:** Dietary iron sufficiency among nonpregnant adults with iron deficiency anemia.

## Data Availability

Computer code used to generate the study results is be posted to the corresponding author's GitHub repository at https://github.com/rvajravelu/idaGiRiskFactors with a README file describing the function of each file. These files are freely available. All raw data are freely available via the National Center for Health Statistics at https://www.cdc.gov/nchs/nhanes.
