# Supplementary material for: Distribution of Gastrointestinal and Dietary Risk Factors Among U.S. Adults Classified as Having Iron Deficiency Anemia Across Diagnostic Thresholds
Source: Am J Hematol. 2026 May 30;101(8):2095–9. doi: 10.1002/ajh.70388 (PMC13331639; doi:10.1002/ajh.70388)
Supplement: Supplementary file 1 — Table S1: Unweighted counts. Figure S1: Gastrointestinal risk factors among nonpregnant adults with iron deficiency anemia stratified by sex and menopausal status. Figure S2: Age distribution among nonpregnant adults with iron deficiency anemia. Figure S3: Dietary iron sufficiency among nonpregnant adults with iron deficiency anemia. [file AJH-101-2095-s001.docx]

**Supplemental Material**

Distribution of Gastrointestinal and Dietary Risk Factors Among U.S. Adults Classified as Having Iron Deficiency Anemia Across Diagnostic Thresholds

Omar Al Ta’ani MD*, Bridget M. Mayrer MS*, Nicole M. Luche MD, Shazia Mehmood Siddique MD MS, Scott A. Peslak MD PhD, Timothy S. Anderson MD MAS, Ravy K. Vajravelu MD MSCE

**Contents (hyperlinks):**

[Supplemental Methods 2](#_Toc229143639)

[Supplemental References 7](#_Toc229143640)

[Data Dictionary 9](#_Toc229143641)

[Supplemental Table 1. Unweighted counts 12](#_Toc229143642)

[Supplemental Figure 1. Gastrointestinal risk factors among non-pregnant adults with iron deficiency anemia stratified by sex and menopausal status. 13](#_Toc229143643)

[Supplemental Figure 2. Age distribution among non-pregnant adults with iron deficiency anemia. 14](#_Toc229143644)

[Supplemental Figure 3. Dietary iron sufficiency among non-pregnant adults with iron deficiency anemia. 15](#_Toc229143645)

# Supplemental Methods

*Data Source*

We analyzed data from NHANES, a repeated cross-sectional survey conducted by the Centers for Disease Control and Prevention to assess the health of individuals living in the United States. NHANES includes interviews, physical examinations, and biometric measurements. All protocols are approved by the National Center for Health Statistics Ethics Review Board.^1^ We used data from the 2017 to pre-pandemic 2020 cycle, which is the most recent cycle to have serum ferritin data for pre-menopausal women, post-menopausal women, and men. The 2021 – 2023 cycle was not used as it only contains ferritin data for women aged 12 – 48 and children aged 1 – 5.

*Study Population*

The study population included non-pregnant NHANES participants aged 20 and older to align with the clinical scope of the AGA IDA guideline. Age 20 was selected as the lower age boundary because subsets of NHANES questions and measurements were not conducted among younger participants. NHANES reports gender as male or female, which are usually indicators of biologic sex instead of identity. These gender data are collected from participant self-report without clarification of whether it considers sex assigned at birth. Therefore, we interpreted the gender variable as a proxy for sex in this study, but we use the terms “men” and “women” to refer to the study participants instead of “males” and “females” as the self-report nature of the gender variable implies that it refers to identity. Pregnancy status was based on urine pregnancy test. Because abnormal uterine bleeding is a common cause of iron deficiency, we stratified participants by sex, and women were sub-stratified as pre- or post-menopausal. Women were classified as post-menopausal if they stated they had not had a menstrual period in the prior 12 months due to menopause

*Handling of missing data and determination of menopause status*

After accounting for survey weights, there were missing data in three of the study concepts: date of last menstrual period, ferritin, and advanced hepatic fibrosis/cirrhosis. Fourteen and seven-tenths percent of women participants had missing menstrual period data. To classify these individuals as pre-menopausal or post-menopausal, we estimated the population-level median age of menopause among post-menopausal women who reported their age at last menstrual period. Using the interview weight, the median age at menopause among post-menopausal women was 48. Women participants with missing menstrual period data younger than 48 were classified as pre-menopausal and women participants with missing menstrual period data age 48 and older were classified as post-menopausal. Additionally, women who reported hysterectomy were classified with post-menopausal women due to similar risk of uterine blood loss. Finally, women who had not reported menopause beyond the outlier age of the third quartile plus 1.5 times the interquartile range (IQR) were classified as post-menopausal. The outlier menopause age was 70. Thirteen and six-tenths percent of participants had missing ferritin data. NHANES analytic guidance describes that 95% of adult participants provided blood samples and that missing data is due to too small phlebotomy samples to complete the over 800 conducted examinations. As such, we considered missing ferritin data to be missing at random. To account for this missingness, we conducted all analyses involving iron deficiency among the subpopulation of non-pregnant adult participants who had available ferritin data. Additionally, outlier ferritin values higher than the third quartile plus 1.5 times the IQR were excluded to avoid skewing the distribution by iron overload conditions. The outlier ferritin level was 398 ng/ml. Fifteen and one-tenth percent of participants were missing at least one component of the FIB-4 score used to assess advanced hepatic fibrosis/cirrhosis. As described above for missing ferritin values, we considered these to be missing at random and excluded individuals with missing FIB-4 scores from analyses that included gastrointestinal risk factors

*IDA Definitions*

Iron deficiency was classified using three ferritin thresholds: ≤15 ng/ml, consistent with WHO recommendations, ≤30 ng/ml, consistent with the 2025 ASH draft IDA guideline, and ≤45 ng/ml, consistent with the 2020 AGA IDA guideline.^2-7^ Anemia was defined by WHO criteria as hemoglobin ≤12 g/dL for women and ≤13 g/dL for men.^8^ IDA was classified as the presence of iron deficiency and anemia.

*IDA Risk Factors*

Because the AGA guideline recommends evaluating individuals with IDA for gastrointestinal etiologies via bidirectional endoscopy, we assessed gastrointestinal risk factors among patients with IDA. Gastrointestinal bleeding risk factors included congestive heart failure and chronic kidney disease due to their association with arteriovenous malformations, advanced hepatic fibrosis/cirrhosis due to its association with portal hypertensive gastropathy, and anticoagulant, antiplatelet, aspirin, or non-steroidal anti-inflammatory drug (NSAID) use due to their potential adverse drug effect of gastrointestinal hemorrhage.^9-14^ Medical conditions and medications were ascertained from the Medical Conditions and Prescription Medications questionnaires, respectively.

We also assessed abdominal pain without gastrointestinal bleeding risk factors. Abdominal pain is a cardinal symptom of gastrointestinal conditions that can cause iron deficiency such as celiac disease, inflammatory bowel disease, and *Helicobacter pylori* infection. As such, in clinical settings, patients with IDA experiencing abdominal pain may be considered for gastrointestinal evaluation. We identified abdominal pain from the Medical Conditions questionnaire through endorsement of non-menstrual abdominal pain in the past 12 months. Whether the abdominal pain was non-menstrual or menstrual was assessed by the NHANES participant and interviewer through visual aids that localize history of pain to the abdomen instead of the pelvis. Individuals with history of gastrointestinal malignancy were excluded from the gastrointestinal bleeding risk factors and abdominal pain analyses because the cancers would represent a known, rather than occult, source of gastrointestinal bleeding.

Because occult gastrointestinal bleeding could be caused by undiagnosed colorectal cancer, we assessed the age distribution of individuals with IDA. The age categories were 20 – 44 and ≥45 based on the age of average-risk colorectal cancer screening initiation from the 2020 U.S. Preventative Services Task Force Screening for Colorectal Cancer Guidelines and the 2022 U.S. Multisociety Task Force on Colorectal Cancer update.^15,16^

To assess the proportion of iron-deficient individuals who could initially be treated with iron supplementation without additional evaluation, we assessed iron intake from the average of the day one and day two 24-hour dietary recall and dietary supplement interviews. Dietary iron sufficiency was classified based on Dietary Reference Intakes from the National Academies.^17^

*Statistical Analysis*

At each ferritin threshold, we calculated the number of individuals with IDA meeting criteria for each of the measures described above, stratified by sex and menopause status. Because NHANES is a probabilistic survey, all estimates were weighted using primary sampling units and masked variance pseudostrata to generalize the results to the U.S. population.^18^ Most analyses utilized the mobile examination center weights because non-fasting laboratory data was used to identify iron deficiency. Analyses with dietary data used the dietary day two sample weight, which is a subset of the mobile examination center weight. Analyses were conducted using appropriate survey and subpopulation commands. To calculate the number of individuals newly classified as having IDA stratified by sex, menopause status, and the IDA risk factors described above, we used bootstrapping to calculate 95% CIs instead of propagation of standard errors because the survey subpopulations for individuals with IDA at each threshold are not statistically independent. Bootstrapping was performed by resampling participants within strata with replacement to preserve validity of the weighted estimates and variances based on the complex, stratified NHANES survey design. Data extraction, cleaning, and analyses were performed using R (version 4.3.2) and its *survey* package (version 4.4-2).^19,20^

# Supplemental References

1. NCHS Ethics Review Board (ERB) Approval. National Center for Health Statistics. Accessed 09/10/2024, <https://www.cdc.gov/nchs/nhanes/irba98.htm>

2. Ko CW, Siddique SM, Patel A, et al. AGA Clinical Practice Guidelines on the Gastrointestinal Evaluation of Iron Deficiency Anemia. *Gastroenterology*. Sep 2020;159(3):1085-1094. doi:10.1053/j.gastro.2020.06.046

3. Rockey DC, Altayar O, Falck-Ytter Y, Kalmaz D. AGA Technical Review on Gastrointestinal Evaluation of Iron Deficiency Anemia. *Gastroenterology*. Sep 2020;159(3):1097-1119. doi:10.1053/j.gastro.2020.06.045

4. *WHO guideline on use of ferritin concentrations to assess iron status in individuals and populations*. 2020. Accessed 09/10/2024. <https://iris.who.int/bitstream/handle/10665/331505/9789240000124-eng.pdf?sequence=1&isAllowed=y>

5. Ning S, Zeller MP. Management of iron deficiency. *Hematology Am Soc Hematol Educ Program*. Dec 6 2019;2019(1):315-322. doi:10.1182/hematology.2019000034

6. Iolascon A, Andolfo I, Russo R, et al. Recommendations for diagnosis, treatment, and prevention of iron deficiency and iron deficiency anemia. *Hemasphere*. Jul 2024;8(7):e108. doi:10.1002/hem3.108

7. Iron Deficiency Anemia Project Plan. American Society of Hematology. Accessed 10/21/2025, <https://www.hematology.org/education/clinicians/guidelines-and-quality-care/clinical-practice-guidelines/iron-deficiency-anemia>

8. *Guideline on haemoglobin cutoffs to define anaemia in individuals and populations*. World Health Organization; 2024. Accessed 09/10/2024. <https://iris.who.int/bitstream/handle/10665/376196/9789240088542-eng.pdf?sequence=1>

9. Tariq T, Karabon P, Irfan FB, et al. Secondary angiodysplasia-associated gastrointestinal bleeding in end-stage renal disease: Results from the nationwide inpatient sample. *World J Gastrointest Endosc*. Oct 16 2019;11(10):504-514. doi:10.4253/wjge.v11.i10.504

10. Randi AM, Laffan MA, Starke RD. Von Willebrand factor, angiodysplasia and angiogenesis. *Mediterr J Hematol Infect Dis*. Sep 2 2013;5(1):e2013060. doi:10.4084/MJHID.2013.060

11. Khalifa A, Rockey DC. Role of Endoscopy in the Diagnosis, Grading, and Treatment of Portal Hypertensive Gastropathy and Gastric Antral Vascular Ectasia. *Gastrointest Endosc Clin N Am*. Apr 2024;34(2):263-274. doi:10.1016/j.giec.2023.09.013

12. Bermont A, Abu-Freha N, Cohen DL, et al. Epidemiology and risk factors for angiodysplasias of the upper and lower gastrointestinal tract: A large population-based study. *Dig Liver Dis*. Aug 17 2024;doi:10.1016/j.dld.2024.07.037

13. Abraham NS, Noseworthy PA, Yao X, Sangaralingham LR, Shah ND. Gastrointestinal Safety of Direct Oral Anticoagulants: A Large Population-Based Study. *Gastroenterology*. Apr 2017;152(5):1014-1022 e1. doi:10.1053/j.gastro.2016.12.018

14. Abraham NS, Cohen DC, Rivers B, Richardson P. Validation of administrative data used for the diagnosis of upper gastrointestinal events following nonsteroidal anti-inflammatory drug prescription. *Aliment Pharmacol Ther*. Jul 15 2006;24(2):299-306. doi:10.1111/j.1365-2036.2006.02985.x

15. Davidson KW, Barry MJ, Mangione CM, et al. Screening for Colorectal Cancer: US Preventive Services Task Force Recommendation Statement. *JAMA*. May 18 2021;325(19):1965-1977. doi:10.1001/jama.2021.6238

16. Patel SG, May FP, Anderson JC, et al. Updates on Age to Start and Stop Colorectal Cancer Screening: Recommendations From the U.S. Multi-Society Task Force on Colorectal Cancer. *Gastroenterology*. 2022/1// 2022;162(1):285-299. doi:10.1053/J.GASTRO.2021.10.007/ASSET/45363639-86F0-46DC-A233-B1838881688E/MAIN.ASSETS/GR1.JPG

17. *Dietary Reference Intakes*. 2006.

18. Akinbam L, Chen T-C, Davy O, et al. 2022;doi:10.15620/cdc:115434

19. *R: A language and environment for statistical computing. R Foundation for Statistical Computing*. R Core Team; 2020. <https://www.R-project.org>

20. *survey: Analysis of Complex Survey Samples*. Version 4.4-2. 2024. <https://cran.r-project.org/web/packages/survey/index.html>

# Data Dictionary

See <https://wwwn.cdc.gov/nchs/nhanes/continuousnhanes/default.aspx?Cycle=2017-2020> for additional details on each variable.

| **NHANES Variable** | **NHANES Dataset** | **Variable description** | **Question to participant** | **Target** |
| --- | --- | --- | --- | --- |
| LBXSASSI | P_BIOPRO | Aspartate Aminotransferase (AST) (U/L) |  | Both males and females 12 YEARS - 150 YEARS |
| LBXSATSI | P_BIOPRO | Alanine Aminotransferase (ALT) (U/L) |  | Both males and females 12 YEARS - 150 YEARS |
| LBXHGB | P_CBC | Hemoglobin (g/dL) |  | Both males and females 1 YEARS - 150 YEARS |
| LBXPLTSI | P_CBC | Platelet count (1000 cells/uL) |  | Both males and females 1 YEARS - 150 YEARS |
| RIAGENDR | P_DEMO | Gender | Gender of the participant. | Both males and females 0 YEARS - 150 YEARS |
| RIDAGEYR | P_DEMO | Age at Screening Adjudicated | Age in years of the participant at the time of screening. Individuals 80 and over are topcoded at 80 years of age. | Both males and females 0 YEARS - 150 YEARS |
| RIDRETH3 | P_DEMO | Race/Hispanic origin w/ NH Asian | Recode of reported race and Hispanic origin information, with Non-Hispanic Asian Category | Both males and females 0 YEARS - 150 YEARS |
| SDMVPSU | P_DEMO | Masked variance pseudo-PSU | Masked variance unit pseudo-PSU variable for variance estimation | Both males and females 0 YEARS - 150 YEARS |
| SDMVSTRA | P_DEMO | Masked variance pseudo-stratum | Masked variance unit pseudo-stratum variable for variance estimation | Both males and females 0 YEARS - 150 YEARS |
| SEQN | P_DEMO | Respondent sequence number |  | Both males and females 0 YEARS - 150 YEARS |
| WTINTPRP | P_DEMO | Full sample interview weight |  | Both males and females 0 YEARS - 150 YEARS |
| WTMECPRP | P_DEMO | Full sample MEC exam weight |  | Both males and females 0 YEARS - 150 YEARS |
| DR1TIRON | P_DR1TOT | Iron (mg) | Total dietary iron intake from dietary recall day one interview | Both males and females 0 YEARS - 150 YEARS |
| WTDRD1PP | P_DR1TOT | Dietary day one sample weight |  | Both males and females 0 YEARS - 150 YEARS |
| DR2TIRON | P_DR2TOT | Iron (mg) | Total dietary iron intake from dietary recall day two interview | Both males and females 0 YEARS - 150 YEARS |
| WTDR2DPP | P_DR2TOT | Dietary two-day sample weight |  | Both males and females 0 YEARS - 150 YEARS |
| DS1IIRON | P_DS1IDS | Iron (mg) | Supplemental iron intake from dietary recall day one interview | Both males and females 0 YEARS - 150 YEARS |
| DS2IIRON | P_DS1IDS | Iron (mg) | Supplemental iron intake from dietary recall day two interview | Both males and females 0 YEARS - 150 YEARS |
| DSD090 | P_DSQIDS | How long supplement taken (days)? | For how long have you been taking {PRODUCT NAME} or a similar type of product? | Both males and females 0 YEARS - 150 YEARS |
| DSDSUPP | P_DSQIDS | Supplement Name |  | Both males and females 0 YEARS - 150 YEARS |
| DSQ124 | P_DSQIDS | Took product on own or doctor advised | What is the reason you take {PRODUCT NAME}? (Did you decide to take it for reasons of your own or did a doctor or other health provider tell you to take it?) | Both males and females 0 YEARS - 150 YEARS |
| DSQ128R | P_DSQIDS | For anemia, such as low iron | For what reason or reasons do you take {PRODUCT NAME}? (For what reason or reasons did the doctor or other health professional tell you to take {PRODUCT}?) | Both males and females 0 YEARS - 150 YEARS |
| LBXFER | P_FERRITIN | Ferritin (ug/L) |  | Both males and females 1 YEARS - 5 YEARS, Both males and females 12 YEARS - 150 YEARS |
| HIQ011 | P_HIQ | Covered by health insurance | {Are you/Is SP} covered by health insurance or some other kind of health care plan? [Include health insurance obtained through employment or purchased directly as well as government programs like Medicare and Medicaid that provide medical care or help pay medical bills.] | Both males and females 0 YEARS - 150 YEARS |
| HUQ030 | P_HUQ | Routine place to go for healthcare | Is there a place that {you/SP} usually {go/goes} when {you are/he/she is} sick or {you/s/he} need{s} advice about {your/his/her} health? | Both males and females 0 YEARS - 150 YEARS |
| KIQ022 | P_KIQ_U | Ever told you had weak/failing kidneys; 1=Yes, 2= No | {Have you/Has SP} ever been told by a doctor or other health professional that {you/s/he} had weak or failing kidneys? Do not include kidney stones, bladder infections, or incontinence. | Both males and females 20 YEARS - 150 YEARS |
| MCQ053 | P_MCQ | Taking treatment for anemia/past 3 mos | During the past 3 months, {have you/has SP} been on treatment for anemia (a-nee-me-a), sometimes called "tired blood" or "low blood"? [Include diet, iron pills, iron shots, transfusions as treatment.] | Taking treatment for anemia/past 3 mos |
| MCQ092 | P_MCQ | Ever receive blood transfusion; | {Have you/Has SP} ever received a blood transfusion? | Both males and females 6 YEARS - 150 YEARS |
| MCQ160B | P_MCQ | Ever told had congestive heart failure | Has a doctor or other health professional ever told {you/SP} that {you/s/he} . . .had congestive heart failure? | Both males and females 20 YEARS - 150 YEARS |
| MCQ230A | P_MCQ | 1st cancer - what kind was it? | 1st cancer - what kind was it? | Both males and females 20 YEARS - 150 YEARS |
| MCQ230B | P_MCQ | 2nd cancer - what kind was it? | 2nd cancer - what kind was it? | Both males and females 20 YEARS - 150 YEARS |
| MCQ230C | P_MCQ | 3rd cancer - what kind was it? | 3rd cancer - what kind was it? | Both males and females 20 YEARS - 150 YEARS |
| MCQ520 | P_MCQ | Abdominal pain during past 12 months? | During the past 12 months {have you/has s/he} had pain in the abdominal area shaded on the diagram? | Both males and females 20 YEARS - 150 YEARS |
| RHD043 | P_RHQ | Reason not having regular periods | What is the reason that {you have/SP has} not had a period in the past 12 months? | Females only 12 YEARS - 150 YEARS |
| RHQ031 | P_RHQ | Had regular periods in past 12 months | {Have you/Has SP} had at least one menstrual period in the past 12 months? (Please do not include bleedings caused by medical conditions, hormone therapy, or surgeries.) | Females only 12 YEARS - 150 YEARS |
| RHQ060 | P_RHQ | Age at last menstrual period (in years) | About how old {were you/was SP} when {you/SP} had {your/her} last menstrual period? | Females only 12 YEARS - 150 YEARS |
| RXDRUG | P_RXQ_RX | Generic drug name |  | Both males and females 0 YEARS - 150 YEARS |
| RXDUSE | P_RXQ_RX | Taken prescription medicine, past month | In the past 30 days, have you used or taken medication for which a prescription is needed? Do not include prescription vitamins or minerals you may have already told me about. | Both males and females 0 YEARS - 150 YEARS |
| RXQ510 | P_RXQASA | Dr told to take daily low-dose aspirin | Doctors and other health care providers sometimes recommend that {you take/SP takes) a low-dose aspirin each day to prevent heart attacks, strokes, or cancer. {Have you/Has SP} ever been told to do this? | Both males and females 40 YEARS - 150 YEARS |
| RXQ515 | P_RXQASA | Followed advice, took low-dose aspirin | {Are you/Is SP} now following this advice? | Both males and females 40 YEARS - 150 YEARS |
| RXQ520 | P_RXQASA | Taking low-dose aspirin on your own | On {your/SP's} own, {are you/is SP} now taking a low-dose aspirin each day to prevent heart attacks, strokes, or cancer? | Both males and females 40 YEARS - 150 YEARS |
| URXPREG | P_UCPREG | Urine Pregnancy Result |  | Females only 20 YEARS - 44 YEARS |

#

# Supplemental Table 1. Unweighted counts

|  | **All non-pregnant  adults** | **Pre-menopausal women** | **Post-menopausal women** | **Men** |
| --- | --- | --- | --- | --- |
| Unweighted n | 7,357 | 1,901 | 2,039 | 3,417 |
| **Demographics** |  |  |  |  |
| Race |  |  |  |  |
| *Mexican American* | 884 | 287 | 190 | 407 |
| *Non-Hispanic Asian* | 832 | 261 | 223 | 348 |
| *Non-Hispanic Black* | 1,862 | 499 | 523 | 840 |
| *Non-Hispanic White* | 2,635 | 553 | 806 | 1,276 |
| *Other Hispanic* | 791 | 202 | 228 | 361 |
| *Other Race/Multi-Racial* | 353 | 99 | 69 | 185 |
| Covered by health insurance | 6,150 | 1,516 | 1,847 | 2,787 |
| Routine place to go for healthcare | 6,154 | 1,570 | 1,895 | 2,689 |
| **Medical history and comorbidities** |  |  |  |  |
| History of blood transfusion | 846 | 144 | 399 | 303 |
| Advance fibrosis/cirrhosis, congestive heart failure, or chronic kidney disease | 564 | 49 | 206 | 309 |
| Anticoagulant, antiplatelet, aspirin, or NSAID use | 1,501 | 86 | 605 | 810 |

# Supplemental Figure 1. Gastrointestinal risk factors among non-pregnant adults with iron deficiency anemia stratified by sex and menopausal status.


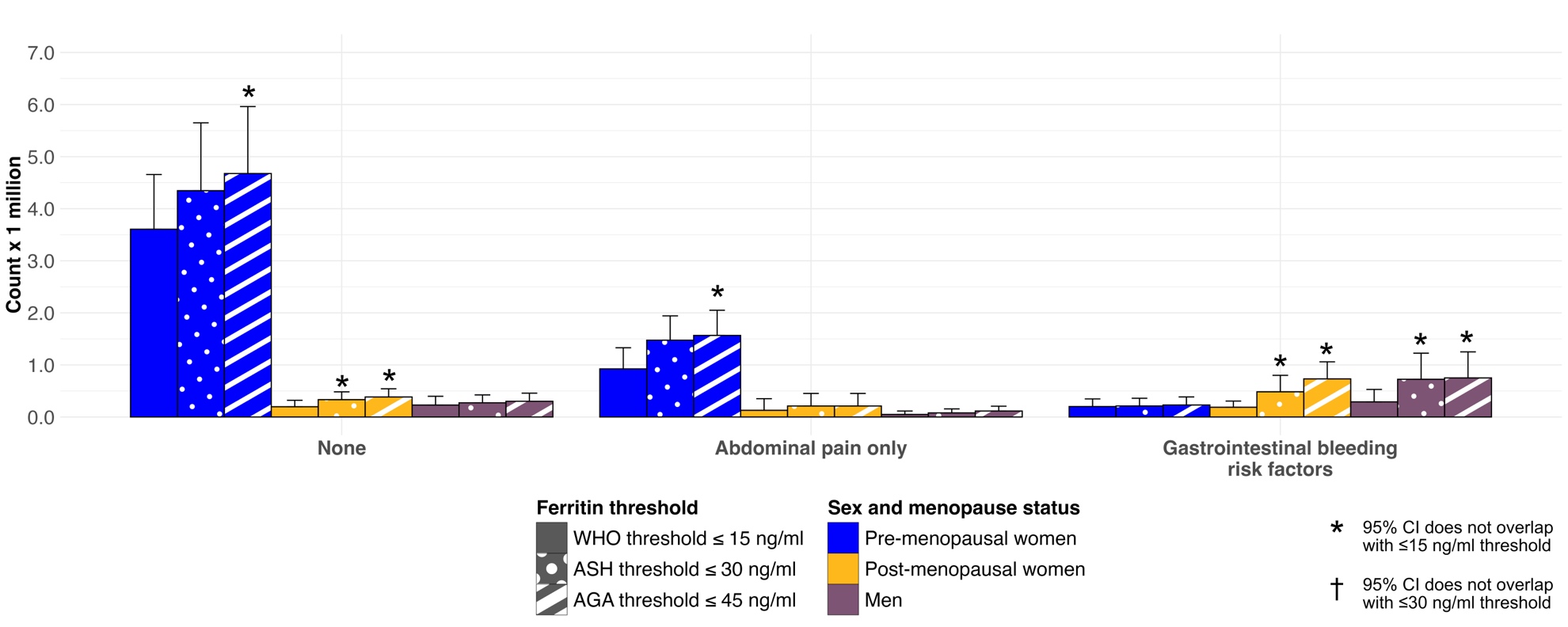


Gastrointestinal bleeding risk factors were congestive heart failure, chronic kidney disease, advanced hepatic fibrosis/cirrhosis, and anticoagulant, antiplatelet, aspirin, and non-steroidal anti-inflammatory drug use. Abdominal pain was ascertained from the medical questionnaire and indicates that the participant had non-menstrual abdominal pain within the past 12 months. Error bars denote 95% CIs.

# Supplemental Figure 2. Age distribution among non-pregnant adults with iron deficiency anemia.

**
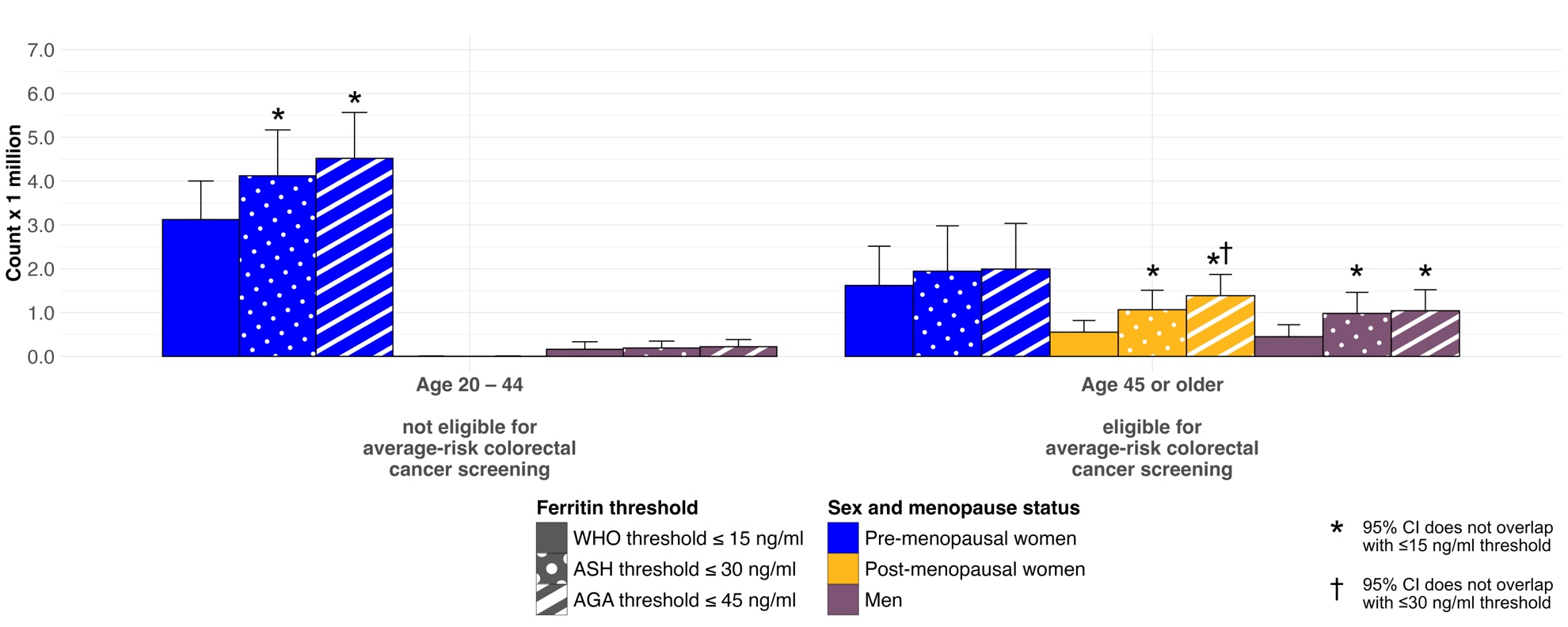
**

Individuals age 45 and older are eligible for average-risk colorectal cancer screening.^15,16^ Error bars denote 95% CIs.

# Supplemental Figure 3. Dietary iron sufficiency among non-pregnant adults with iron deficiency anemia.


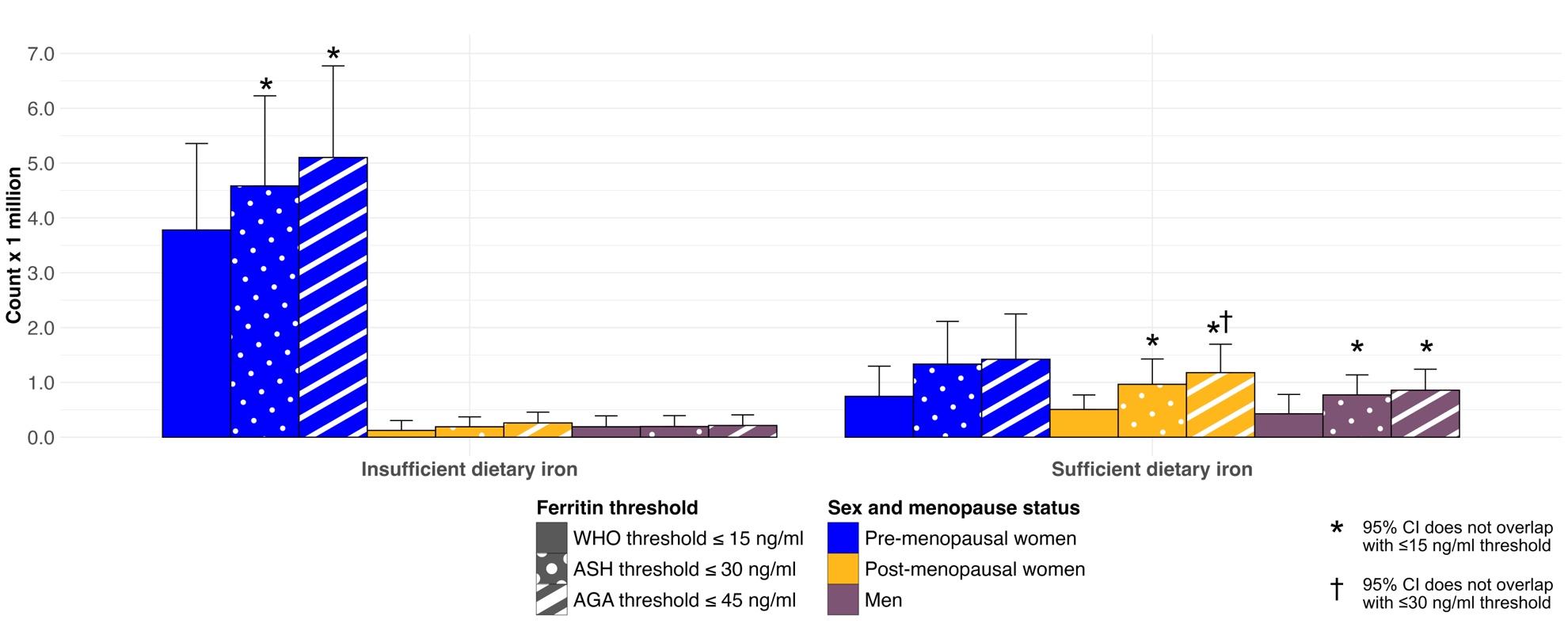


Dietary iron intake was calculated from the average of the day one and day two 24-hour dietary recall interviews plus any iron supplementation. Among non-pregnant adults, median daily iron intake was 11.7 mg (IQR 8.8 – 16.3) for pre-menopausal women (Dietary Reference Intake 18 mg), 11.3 mg (IQR 8.3 – 15.7) for post-menopausal women (Dietary Reference Intake 8 mg), and 14.6 mg (IQR 10.6 – 19.9 for men (Dietary Reference Intake 8 mg). Error bars denote 95% CIs.
